# Temporomandibular joint dysfunction in relation to tinnitus in Peruvian patients: A cross-sectional study

**DOI:** 10.4317/jced.61358

**Published:** 2024-04-01

**Authors:** Toshi-Hiromi Miyamoto-Aldave, Angel-Steven Asmat-Abanto, Delia-Margarita Ulloa-Cueva, Carlos-Alberto Minchón-Medina, Víctor-Eduardo Llanos-Vera, Jannette-Vanessa Callirgos-Briones

**Affiliations:** 1 Student of Stomatology Study Program - Antenor Orrego Private University (Trujillo, Peru); 2 Doctor in Stomatology. Specialist in Periodontics. Professor of Human Medicine Study Program - Antenor Orrego Private University (Trujillo, Peru). Professor of Stomatology Study Program - Antenor Orrego Private University (Trujillo, Peru); 3 Professor of Human Medicine Study Program - Antenor Orrego Private University (Trujillo, Peru). Otorhinolaryngologist at the Víctor Lazarte Echegaray Hospital – Essalud (Trujillo, Peru); 4 Professor of Faculty of Physical Sciences and Mathematics, Department of Statistics, Trujillo National University (Trujillo, Peru); 5 Master in Stomatology. Professor of Stomatology Study Program - Antenor Orrego Private University (Trujillo, Peru); 6 Master in Education E-Learning. Professor of Translation and Interpreting Program, School of Education and Languages, Faculty of Law and Humanities, Cesar Vallejo University (Lima, Peru)

## Abstract

**Background:**

To determine the relationship between temporomandibular joint dysfunction (TMJD) and tinnitus in Peruvian adult patients.

**Material and Methods:**

This observational and cross-sectional study was conducted between April and May 2023, including 76 adult patients from the Otorhinolaryngology Service of Víctor Lazarte Echegaray Essalud Hospital in Trujillo (Peru). The intra- and inter-rater reliability was determined for the clinical measurement of TMJD, obtaining Kappa values above 0.995. To diagnose tinnitus, we worked with a doctor specializing in otolaryngology. Chi-square test and logistic regression were used to analyze results, considering a significance level of *p*<0.05.

**Results:**

A relationship was found between TMJD and tinnitus (*p*=0.022), increasing the frequency of this disorder as temporomandibular involvement was higher (*p*=0.043). There was no relation between these disorders according to gender and age, nor in hypertensive patients (*p*=0.131) or patients suffering from migraine (*p*=0.147); however, a relationship was found between TMJD and tinnitus in patients with hearing loss (*p*=0.046).

**Conclusions:**

TMJD is associated with tinnitus in otorhinolaryngological and hypoacusis patients. However, in hypertensive and migraine patients, and according to gender and age, no relation was found between those disorders.

** Key words:**Tinnitus, Temporomandibular Joint Disorders, Hearing Loss, Sensorineural, Audiometry, Migraine, Hypertension, Headache.

## Introduction

Temporomandibular joint dysfunction (TMJD), also known as temporomandibular disorder (1), includes a group of disorders of the stomatognathic system that affect the temporomandibular joint (TMJ) and usually cause functional limitations (2,3). This dysfunction is evaluated through physical examination, paying special attention to its location, type of pain, irradiation, duration, associated symptoms, and limitation of movements (maximum opening, protrusion, straight path, maximum laterality), which allows for determining the type and origin of the TMJD (4).

The highest incidence of TMJD is evident in adults aged 20 to 40 years, in which women are four times more likely to be affected by the disorder; however, the reported prevalence of symptomatology that requires treatment only occurs in 5 to 12% of the population (2). On the other hand, the literature indicates that TMJD is associated with work stress overload (5), competitive sports, or even dangerous habits where the main risk factor is bruxism (6,7).

Early diagnosis and treatment of TMJD allow for a favorable prognosis. For this reason, if signs or symptoms related to TMJD are recognized during periodic check-ups, the first step of the dentist is to identify a possible causality and inform the patient about the efficient ways of care (1) and the benefits that timely treatment would present (8). It is important to mention that the majority of affected people also have a tendency to report otologic symptoms, including ear pain, vertigo, dizziness, subjective hearing impairment, and tinnitus, the latter being the most prevalent (9).

Tinnitus, derived from the Latin word “tinnire” which means to ring like a bell, includes auditory manifestations that constitute the response of the central nervous system, limbic system, and autonomic system activity (9,10).

There is etiological diversity, so a differential diagnosis should be considered, highlighting the causal factors and associated symptoms (11). This allows for the difference between subjective tinnitus (body noises perceived by the patient as the cardiac system, joint friction and muscle spasms) and objective tinnitus (11,12).

There are three categories of tinnitus: acute tinnitus, which usually lasts less than three months, subacute tinnitus, which lasts 3 to 6 months, and chronic tinnitus, which usually lasts more than 12 months (13). The latter is associated with genetic bases and is very rare, occurring in a population aged between 40 and 54 years (14,15).

Tinnitus can be generated by high levels of stress, resulting in a more intense and distressing sensation, experiencing variations depending on the patient (16,17). Current treatment possibilities include drug therapies and hearing devices (18).

Some studies found that tinnitus is more common in people diagnosed with TMJD (19). The neural interaction between TMJD and tinnitus is based on neural circuits that are largely sensitive to ipsilateral stimuli. This corresponds to the functional connections between the trigeminal spinal nucleus and the cochlear nucleus, both located on the periphery of the major neuronal connections of the brainstem. For this reason, the probable association between tinnitus and TMJD is coherent with a crossover mechanism between the trigeminal system and the cochlear nucleus (20).

Currently, patients come for a consultation with complaints related to tinnitus and TMJ. Therefore, it is important to conduct a personalized study of both the joint and the auditory system, considering anatomically associated factors between them. In this regard, the main objective of this study was to determine the relationship between TMJD and tinnitus in Peruvian adults who underwent audiometry, to provide information about the risk factors of hearing problems, to improve the clinical guidelines of care for the patients, emphasizing the multidisciplinary collaboration between dentists and otolaryngologists. This approach will contribute to improving the quality of life of patients suffering from these pathologies.

## Material and Methods

This study follows a cross-sectional observational design and was conducted at the Víctor Lazarte Echegaray Essalud Hospital in Trujillo (Peru), between April and May 2023.

The sample consisted of 76 patients. It was calculated using the formula for independence tests, based on data generated by a pilot study conducted in 12 people and with the following parameters: α=0.05, β=0.20, r=4 (categories of TMD according to Helkimo Index), c=2 (categories of the presence or absence of tinnitus in patients), pi. (probability of patients with TMD i), p.j (probability of patients with the presence or absence of tinnitus j), pij (joint probabilities of TMD i and presence or absence of tinnitus j), δα,β=10.90256 (non-centrality parameter of the Chi-square distribution with 3 degrees of freedom and the established α and β errors, determined using Minitab 19). The probabilities pi., p.j, and pij, were estimated according to the mentioned pilot study. The sample was selected using the accidental non-probabilistic method.

The included patients were adults over 28 years old who attended an outpatient audiometry examination at the Otorhinolaryngology Service of the Victor Lazarte Echegaray Essalud Hospital. Patients without anterosuperior teeth and those who did not agree to participate in the research were excluded.

This research was conducted with the approval of the Faculty of Human Medicine (Resolution N°0203-2023-FMEHU-UPAO), and Bioethics Committee of the Antenor Orrego Private University (Resolution Bioethics Committee N°N°0116-2023-UPAO), and The Training Directorate of the La Libertad Assistance Network – ESSALUD (RI N°77 CIYE-O.C.I.Y. D-RALL-ESSALUD-2023). These operating units monitor strict compliance with the principles established in the Declaration of Helsinki of the World Medical Association and the General Health Law of Peru No. 26842.

Before requesting their participation, all patients received information about the purpose of the research. When they accepted, they were given an informed consent form to read and sign. Subsequently, the audiometry and the diagnosis of TMJD were performed in the hospital’s Otorhinolaryngology Service.

The diagnosis of tinnitus and its intensity in each patient was determined by the co-author of the study, a doctor specialist in otolaryngology, through an audiometry examination using the MAICO MA52 equipment, which recorded values in hertz (Hz) and decibels (dB). After that, without knowing the result of the audiometric evaluation, the principal author evaluated the presence of TMJD according to the Helkimo Index modified by Maglione, where the following criteria were considered into account: limitation in the range of mandibular movement (maximum opening, laterality to the right and left, maximum protrusion), alterations of joint function (movement with the presence of pain, deviation, locking or blockage, determination of joint sounds with stethoscope), presence of pain when performing one or more movements, pain in masticatory muscles and pain in the temporomandibular joint. The results were recorded in the dedicated data collection form, and categorized into mild TMJD, moderate TMJD, and severe TMJD. This instrument also collected basic demographic and covariate information.

The reliability of the method for measuring the Helkimo index was determined by intra- and inter-rater calibration of the principal investigator with an expert professor with a Master’s degree in Stomatology and Coordinator of the Occlusion Chair for 17 years at the Antenor Orrego Private University in Trujillo (Peru). This evaluation was conducted with 12 patients, obtaining intraclass intra-rater correlations of 0.995 and inter-rater correlations of 0.991 and 0.996 (in the first and second measurements compared to the expert, respectively).

Statistical analysis SPSS 26.0 was performed to collect and process data automatically (IBM, Armonk, NY, USA) and show the results in Tables according to the proposed objectives. The relationship between TMJD and tinnitus was determined by the chi-square statistical test of independence of criteria, and the odds ratio (OR) was reported. Also, the relationship between the primary and secondary variables was assessed using binary logistic regression. The significance level of the tests was *p*<0.05.

## Results

In this research, 76 patients who attended an audiometry examination at the Otorhinolaryngology Service of the Víctor Lazarte Echegaray Essalud Hospital, were evaluated between April and May 2023; 35 (46.7%) patients were female and 41 (53.3%) patients were male, 18 (23.7%) were aged 60 years old, 43 (53.6%) from 61 to 80 years old, and 15 (19.7%) were over 80 years old. The age of the patients ranged from 42 to 95 years, with a mean age of 69.7 ± 12.8 years.

Regarding the prevalence of TMJD, 69 (90.8%) of the examined patients presented this dysfunction, 35 (46.1%) corresponded to mild TMJD, 26 (34.2%) to moderate TMJD, and 8 (10.5%) to severe TMJD. Also, 42 (55.3%) patients had tinnitus.

The relationship between TMJD and tinnitus is shown in Table 1, revealing a significant association between both disorders (*p* = 0.022). Also, it was found that a higher degree of temporomandibular dysfunction was associated with a greater presence of tinnitus (*p* = 0.043). The presentation of TMJD in patients increases the likelihood of having tinnitus by a factor of 8.8 compared to those without this dysfunction, according to the odds ratio (OR). For mild TMJD, this likelihood is multiplied by 7.1, increasing to 8.2 for moderate dysfunction, and up to 42 times for severe cases.

**Table 1:**
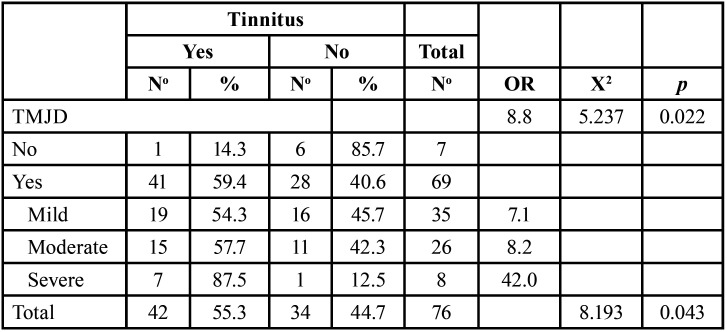
Relationship between temporomandibular joint dysfunction and tinnitus in adult patients attending audiometric examination.

The relationship between gender and age of patients with the presence of tinnitus is shown in Table 2, and there was no significant association between gender and tinnitus (*p*=0.761) nor between age and tinnitus (*p*=0.686) in the evaluated sample. On the other hand, no relationship was found between TMJD and tinnitus (*p*=0.093) according to gender and there was no relationship in patients aged 61 to 80 years (*p*=0.086). There was no association between both disorders in males (*p*=0.107); nor in patients up to 60 years old (*p*=0.444), or in those over 80 years old (*p*=0.467).

**Table 2:**
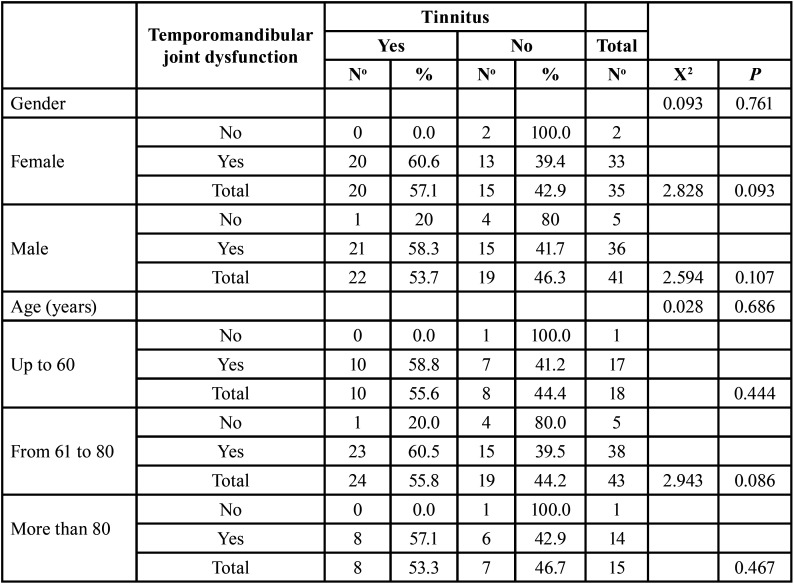
Relationship between temporomandibular joint dysfunction and tinnitus in adult patients attending the audiometric examination, according to gender and age.

Table 3 shows the relationship between TMJD and tinnitus in the presence or absence of other disorders. In this regard, no significant association was found between hearing loss (*p*=0.879), hypertension (*p*=0.595), or migraine (*p*=0.916) with the presence of tinnitus. However, in patients with hearing loss, a relationship between TMJD and tinnitus was found (*p*=0.046), but not in hypertensive patients (*p*=0.131), nor in patients suffering from migraine (*p*=0.147).

**Table 3:**
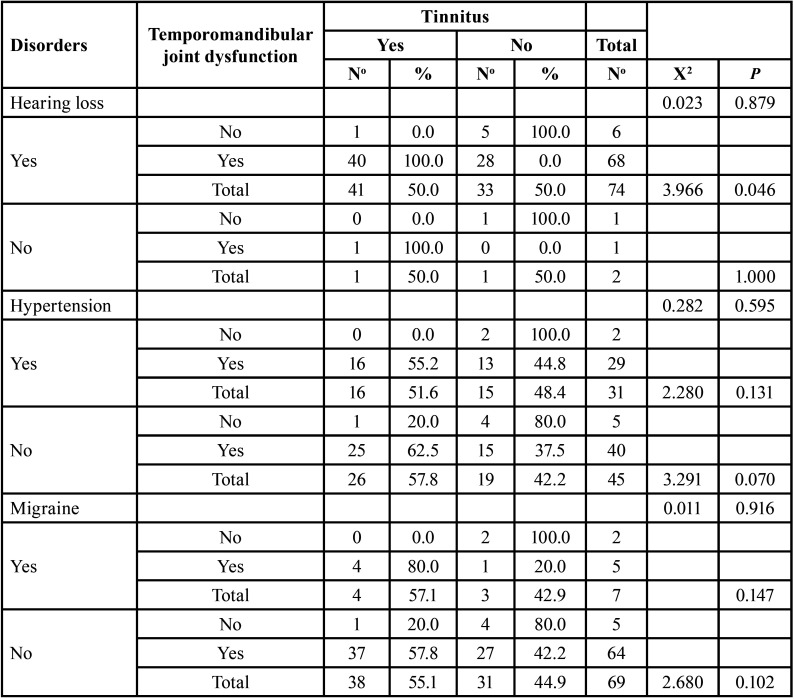
Relationship between temporomandibular joint dysfunction and tinnitus in adult patients attending the audiometric examination, according to disorders.

In addition, the factors under study were analyzed in a multivariate manner using binary logistic regression analysis, as shown in Table 4. First through the presence or absence of TMJD (model 1) and later considering TMJD levels (model 2). According to model 1, the relationship of TMJD with tinnitus (*p* = 0.042) is ratified, multiplying by 9,952 times the possibility of presenting it when you have TMJD. No relationship was found between the other factors considered as covariates, or at least if the patient already has TMJD. Model 2 makes more evident the relationship between TMJD and tinnitus, the possibility of presenting it is multiplied by 8.704 (*p* = 0.063) when the dysfunction is mild, by 10.149 (*p* = 0.0498) when it is moderate, and by 67.809 (*p* = 0.008) when it is severe. And, similar to model 1, no relationship was found between the covariates and the presence of tinnitus.

**Table 4:**
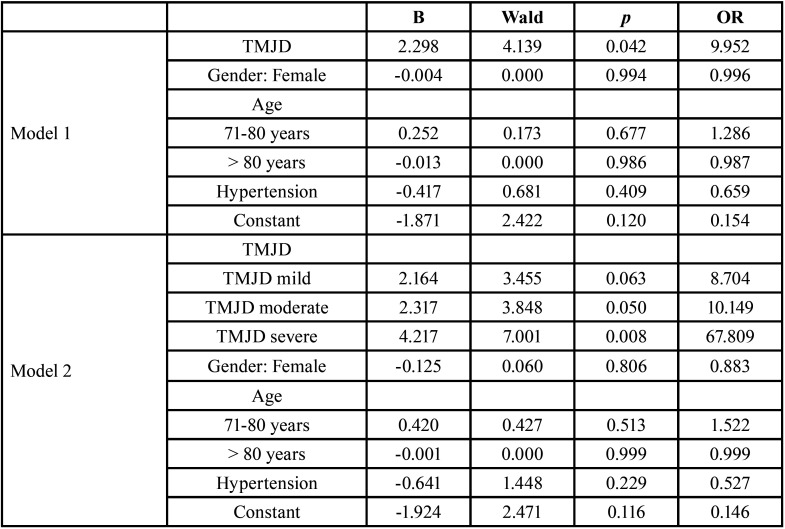
Logistic regression analysis of TMJD and other tinnitus-related factors in adult patients attending the audiometry examination.

## Discussion

Tinnitus is an otologic manifestation (1), that occurs as a response of the central nervous system (9), limbic system, and autonomic system (10). It is characterized by the perception of buzzing or ringing sounds of varying intensities (15). Many of the reviewed studies suggest a possible association with TMJD, which includes disorders of the stomatognathic system directly affecting the TMJ (2), presenting with it a series of functional limitations and symptoms, and requiring a personalized treatment (4).

This research found that tinnitus was associated with the diagnosis of TMJD, considering the presence or absence of this disorder and its levels, finding that the risk of tinnitus is 7.8 more likely in patients with TMJD. Similar results were reported by Bernhardt *et al*. (21) and Lee *et al*. (22) This is probably due to neuronal interaction based on nerve circuits that are mostly sensitive to ipsilateral stimuli, consisting of a crossover mechanism between the trigeminal and cochlear nucleus systems; so much so that according to Buergers *et al*. (23) the risk of tinnitus is 8.37 times higher in patients with TMJD.

According to gender, no relationship was found between TMJD and tinnitus, but there was a trend in female patients, but not in men. Similar results were found by Buergers *et al*. (23) and Lee *et al*. (22) where most of the patients evaluated had a higher risk of suffering from both pathologies, compared to men.

A relationship was found between TMJD and tinnitus in patients diagnosed with sensorineural hearing loss. However, Lee *et al*. (22) reported the opposite, indicating that the alteration of the trigeminal nerve caused by TMJD, produces changes in the dorsal cochlear nucleus, significantly affecting the central auditory pathway and causing sensorineural hearing loss. In this regard, it is suggested to conduct specific research related to TMJD, hearing loss, and tinnitus, because TMJD is also associated with hearing loss. On the other hand, Ramos *et al*. (24) noted that there is a relationship between hearing loss and tinnitus, suggesting that both diseases could be treated simultaneously with a hearing device, which was not demonstrated in this research.

According to the diagnosis of hypertension, no association was found between TMJD and tinnitus, which is consistent with the findings of Ramatsoma and Patrick (25), who found that hypertensive adults had a higher frequency of hearing loss and tinnitus compared to non-hypertensive adults, possibly because of increased blood viscosity generating resistance to blood flow. On the other hand, they noted that high blood pressure can also cause bleeding in arteries within the cochlea, resulting in hearing loss and tinnitus.

According to the diagnosis of migraine, no relationship was found between TMJD and tinnitus. However, García *et al*. (26) found the opposite, and this could be because migraine generates vasospasms in small arterioles of the cochlea and the labyrinth is widely known as one of the main contributing factors for the presence of tinnitus. The difference with Garcia’s study may be due to differences in sample sizes because migraine in the present study was evaluated as a covariate.

It is known that multivariate analysis evaluates the effect of a factor adjusted for the effect of the other factors considered. In this sense, in the present study, controlling for the effect of the other factors, no association was found between gender, age, hypertension, hearing loss, or migraine and tinnitus; however, for TMJD, the risk of tinnitus was found to increase as TMJD becomes more severe, supporting the findings of the bivariate analysis.

This research, being a cross-sectional study, does not present a sequential follow-up of the variables over time, for this reason, subsequent longitudinal studies are required to strengthen or refute these findings and determine causal relationships.

With the findings of this work, the multidisciplinary treatment of patients with tinnitus is suggested, incorporating the contribution of TMJ dental care into the clinical guidelines of their care.

## Conclusions

TMJD is associated with tinnitus in otorhinolaryngological and hypoacusis patients. However, in hypertensive patients, migraine patients, and according to gender and age, no relation was found between those disorders.
